# Long-Term Diabetes Mellitus Is Associated with an Increased Risk of Pancreatic Cancer: A Meta-Analysis

**DOI:** 10.1371/journal.pone.0134321

**Published:** 2015-07-29

**Authors:** Shanshan Song, Baosheng Wang, Xin Zhang, Liliang Hao, Xianliang Hu, Zhongxiang Li, Shaolong Sun

**Affiliations:** Department of Pancreas and Breast Surgery, Shengjing Hospital of China Medical University, Shenyang, Liaoning, China; Leiden University Medical Centre, NETHERLANDS

## Abstract

**Purpose:**

Previous studies have shown a bidirectional relationship between diabetes and pancreatic cancer (PC). In particular, new-onset diabetes might be induced by PC, and people with long-term diabetes might be at increased risk for the development of PC. The purpose of our study was to examine whether long-term diabetes represented an independent risk factor for PC development.

**Methodology:**

A literature search was performed by searching electronic databases for studies published before July 1, 2014, and relative risks (RRs) and corresponding 95% confidence intervals (CIs) were calculated. Data pertaining to diabetes were recorded at both individual and study levels, with RRs calculated separately to analyze the relationship between the duration of diabetes and the development of PC.

**Results:**

Forty-four studies were included in this meta-analysis, including 18 studies with a case-control design, 5 with a nested case-control design and 21 with a cohort design. The overall summary estimate for the relationship between the population with a duration of diabetes ≥2 years and PC was 1.64 (1.52-1.78). The pooled RR (95% CI) of PC for the population with a duration of diabetes ≥5 years was 1.58 (1.42-1.75). For the population with a duration of diabetes ≥10 years, the RR (95% CI) of PC was 1.50 (1.28-1.75).

**Conclusions:**

Our study suggests that long-term diabetes mellitus is associated with an increased risk of PC. However, the level of risk is negatively correlated with increasing diabetes mellitus duration.

## Introduction

Diabetes and cancer are common diseases, the incidences of which are increasing rapidly worldwide. The International Diabetes Federation (IDF) estimated the prevalence of diabetes in adults (20–79 years old) to be 9.6% in 2013, and this number is projected to grow from 8.0% to 9.9% by 2035 in North America and the Caribbean [1]. Furthermore, the IDF expects the prevalence of this disease to reach 9.8% by 2035 in South and Central America [2]. In the European region, the overall prevalence was estimated to be 8.5% in 2013 [3]. Additionally, the WHO has projected that the global cancer incidence will increase from 14 million in 2012 to 22 million in 2032 [4].

Diabetes and cancer have been closely linked epidemiologically and biologically. Convincing evidence has indicated that diabetes (mainly type 2) is associated with an increased risk for several cancers (colorectal, breast, endometrium, liver, pancreas, and bladder) [5] and that diabetes can advance the cancer stage and increase mortality [6–7]. The risk of pancreatic cancer (PC) is increased among individuals with glucose intolerance, including those with excess weight, diabetes mellitus (DM), and high serum glucose. The mechanisms of these associations include the tumorigenic effect of hyperglycemia, the mitogenic effect of obesity-associated hyperinsulinemia and the chronic, subclinical inflammation caused by fat infiltration of the pancreas. However, there has been little direct evidence supporting these mechanisms.

PC is a highly aggressive cancer, with a 5-year overall survival rate of less than 1.0%, and is one of the most frequent causes of cancer deaths worldwide [8]. Surgical resection is considered the best potentially curative treatment for PC, and major advances in this field have successfully lowered the mortality from this disease. However, the impact of surgical resection on overall survival remains minimal, and the local failure rate might remain as high as 50% to 80% in patients who successfully undergo surgical resection, resulting in poor quality of life [9].

Several risk factors such as older age, genetic predisposition, and smoking have been identified as contributors to the burden of PC. Diabetes is also widely considered to be associated with PC, although whether diabetes is a cause or a consequence of PC remains controversial. New-onset diabetes could be a symptom caused by PC development, and people with long-term diabetes might be at increased risk for PC.

Several previously published articles have focused on the incidence and mortality of PC associated with diabetes; however, few studies have clearly demonstrated a relationship between the duration of diabetes and PC. Three meta-analyses showed an association between PC and diabetes [10–12]. First, Batabyal et al. performed an analysis focusing on individual categories of disease duration, including <1, 1–4, 5–9 and ≥10 years. This group found that the risk of PC was greatest early after the diagnosis of DM but that it remained elevated long after the diagnosis; the individual relative risk (RR) ranged from 6.69 at <1 year to 1.36 at 10 years [12]. The other two meta-analyses did not study the association between PC and the duration of diabetes.

The aim of our analysis was to examine the influence of long-term diabetes on PC incidence. If a long-term history of diabetes is an independent risk factor for PC, health professionals and clinical doctors should consider patients with this disease to be at high risk and should strongly encourage PC screening and provide appropriate prevention guidelines.

## Methods

### Search strategy and selection criteria

We searched the PubMed, EMBASE and Web of Science databases using the terms “diabetes mellitus,” “pancreas” and “cancer,” as well as the corresponding individual free terms, to acquire articles that were published before July 1, 2014. Furthermore, we reviewed studies in the references of the retrieved articles to search for additional potentially eligible studies.

The inclusion criteria of the meta-analysis were as follows: (1) the study was defined as a case-control study, a nested case-control study or a cohort study and was published before June 2014; (2) the exposure of interest was diabetes, and the outcome of interest was the incidence of PC; (3) the odds ratios (ORs), RRs, incidence rate ratios (IRRs), hazard ratios (HRs) or standardized incidence rates (SIRs), with their 95% confidence intervals (CIs), were reported; and (4) in case-control studies, the participants were diagnosed with diabetes mellitus at least 2 years before participating in the studies, whereas in nested case-control and cohort studies, the participants with a history of diabetes were followed up for at least 2 years prior to the diagnosis of PC. The exclusion criteria were as follows: (1) publications with incomplete data; (2) studies that only focused on the mortality or survival ratio, with death as the outcome instead of the PC incidence; and (3) the study population recruitment overlapped (in which case we only included the most recent study or the study with the largest number of participants).

### Data extraction

Two investigators independently performed the data extraction using a standardized protocol and a data-recording form, and the extractions were then checked by the other authors. The data were independently examined and adjudicated after being extracted and assessed.

The extracted data included the following information: author, year of publication, region, year in which the study was conducted, age, source and number of subjects, gender, mean number of follow-up years, cases/controls with or without DM, elected diabetes duration, sampling scheme, adjusted factors and adjusted ORs/RRs/SIRs/HRs with 95% CIs.

### Quality assessment

The quality of each study was independently evaluated by two investigators according to the Newcastle-Ottawa Scale (NOS), which comprises three parameters for quality assessment: selection, comparability and exposure for case-control studies; and selection, comparability and outcome for cohort studies [13]. The NOS measurements use four stars for selection: two stars for comparability and three stars for exposure or outcome.

The standards of quality assessment using the NOS in case-control and nested case-control studies are as follows, with each item able to receive one star: 1. Case definition adequate: PC cases with independent diagnosis validation; 2. Representativeness of the cases: consecutive or obviously representative series of PC cases; 3. Selection of controls: controls were from community; 4. Definition of controls: controls had no PC history; 5. Comparability of cases and controls on the basis of the design or analysis with a major factor: study controls for age; 6. Comparability with an additional factor: study controls for any additional factor, for example, sex; 7. Ascertainment of exposure: DM diagnosis with plasma glucose concentration criteria and secure records; 8. Same method of ascertainment for cases and controls: exposure of cases and controls with same ascertainment method; and 9. Non-Response rate: same rate for both groups.

The standards of quality assessment using the NOS in cohort studies are as follows, with each item able to receive one star: 1. Representativeness of the exposed cohort: representative of the average DM incidence in the community from which the group was selected; 2. Selection of the non-exposed cohort: drawn from the same community as the exposed cohort. 3. Ascertainment of exposure: DM diagnosis with plasma glucose concentration criteria and secure records. 4. Outcome not present at start: demonstration that PC was not present at the start of the study. 5. Comparability of cases and controls on the basis of the design or analysis with a major factor: study controls for age. 6. Comparability with an additional factor: study controls for any additional factor, for example, sex. 7. Assessment of outcome: with PC diagnosis certification or record. 8. Follow-up length: follow-up length ≥2 years was considered adequate. 9. Adequacy of follow up: complete follow up that accounted for all subjects or that lost only a small number of subjects.

We defined studies with NOS scores of 1–3 as low quality, those with scores of 4–6 as medium quality, and those with scores of 7–9 as high quality. Studies with discrepancies were simultaneously re-evaluated by the two investigators.

### Statistical analyses

We included different measurements in this meta-analysis, ORs were included for the case-control studies, whereas RRs, IRRs, SIRs and HRs were included for the nested case-control studies and cohort studies. Because of the low worldwide incidence of PC, in practice, these effect measures are assumed to be approximately equivalent to the RR. Therefore, we used the pooled RR and its 95% CI to estimate the risk of PC in long-term DM patients in the present study.

Heterogeneity across the studies was checked with the *Q-*test, which was considered significant when P<0.1. The I^2^ statistic presented the percentage of total variation across studies as a result of heterogeneity. If I^*2*^>50%, obvious heterogeneity was demonstrated, and a random-effect model was used. Otherwise, a fixed-effect model was used to pool the data [14].

Summary estimates of the population diagnosed with DM and followed up for at least 2, 5 and 10 years before participating in the studies were all calculated using a random-effect model.

We conducted sensitivity analysis to investigate the influence of a single study on the overall meta-analysis estimate. Additionally, subgroup analyses that stratified the data separately by gender, study design and region with different DM durations were performed to examine how the strengths of the association varied.

Publication bias was evaluated using Begg’s test [15], and bias was considered to exist when P<0.05. Subsequently, Begg’s funnel plot was created. All of the statistical analyses were performed using Stata software, version 11.0.

## Results

### Study characteristics and quality assessment results

**Fig 1:**
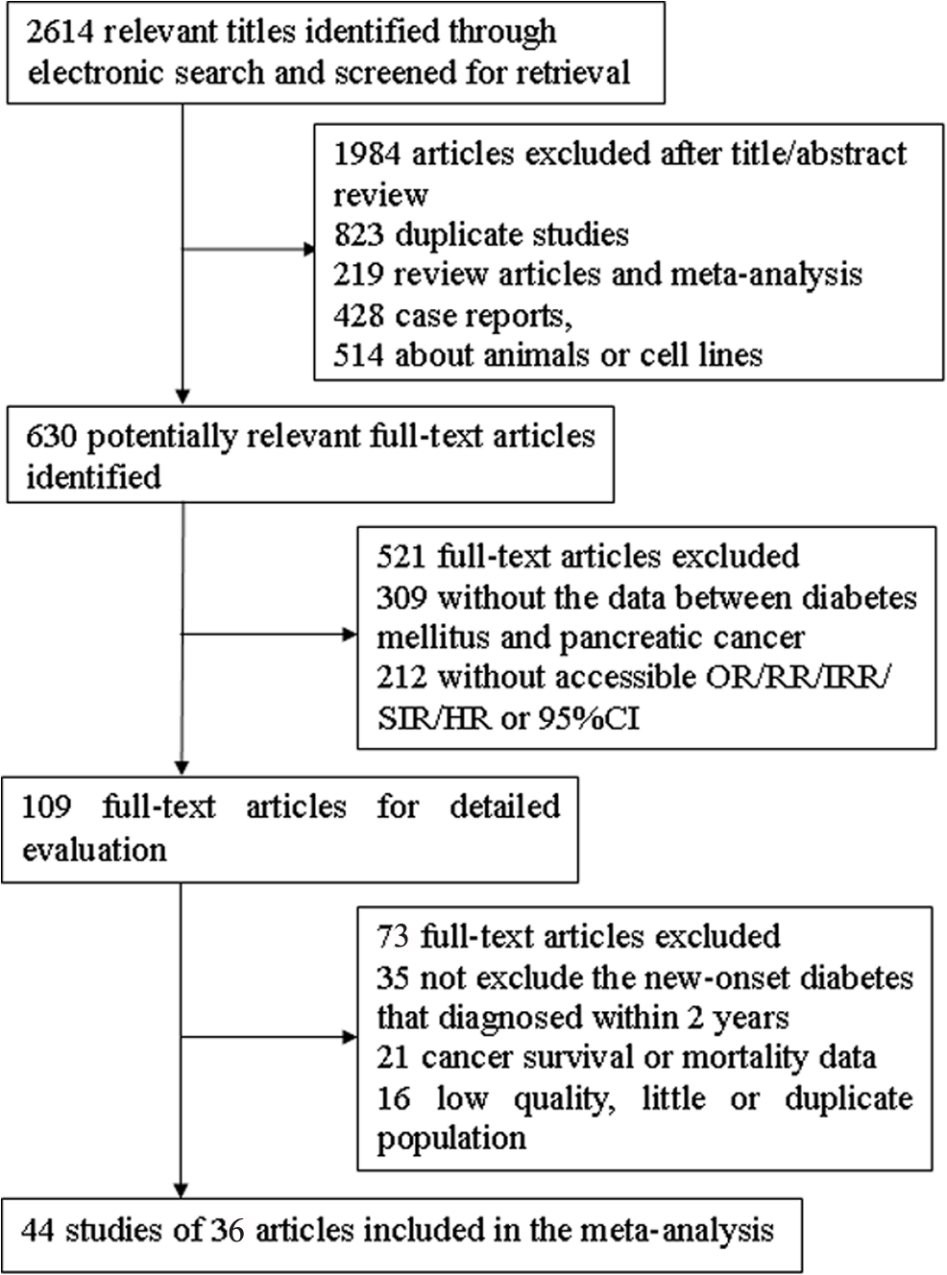
Flow chart showing the study selection process.

We identified 2,614 relevant titles through electronic searches and screened them based on our search criteria, resulting in 630 potentially relevant full-text articles. Of these articles, 44 studies from 36 articles were analyzed (Fig 1). Of the 44 studies included in this meta-analysis, 18 used a case-control design (Table 1), 5 used a nested case-control design (Table 2), and 21 were cohort studies (Table 3).

**Table 1:** Characteristics of 18 case-control studies.

Author/year/region	Age	Gender (% male)	Case (DM with elected duration/ Non-DM), N			Control (DM with elected duration/ Non-DM), N			Elected DM duration, years	Sampling scheme ^a^ [70]		Adjusted factors
			≥2-year	≥5-year	≥10-year	≥2-year	≥5-year	≥10-year		Control selection	Interpretation of reported effect measure	
Cuzick/1989/England [16]	NA	Both (48.5)	9/188	9/188	NA	3/274	3/274	NA	>5	Patients with non-tumor disease, the same hospital and period	Prevalence OR	Age and gender
Farrow/1990/America [17]	20–74	Male	12/156	NA	NA	3/192	NA	NA	≥3	Population	Prevalence OR	Age, smoking and education
Jain/1991/Canada [18]	35–79	Both (54.5)	21/190	21/190	12/190	35/458	35/458	24/458	>5	Population	Prevalence OR	Smoking, calories and fiber intake
Bueno de Mesquita/1992/the Netherlands (M) [19]	35–79	Male	3/85	3/85	NA	12/213	12/213	NA	≥5	Population	Prevalence OR	Age, response status and smoking
Bueno de Mesquita/1992/the Netherlands (F) [19]	35–80	Female	4/64	4/64	NA	13/224	13/224	NA	≥5	Population	Prevalence OR	Age, response status and smoking
Gullo/1994/Italy [20]	22–79	Both (NA)	83/556	61/556	34/556	56/660	36/660	32/660	≥2	Patients with non-tumor disease, the same hospital and period	Prevalence OR	NA
Lee/1996/China [21]	NA	Both (78.7)	54/201	NA	NA	28/247	NA	NA	>3	Physical checkup, the same hospital and period	Prevalence OR	Smoking, alcohol, history of cholecystectomy and gastric resection
Frye/2000/New Zealand [22]	NA	Both (50.0)	9/88	9/88	NA	7/107	7/107	NA	>5	Patients with non-tumor disease, the same hospital and period	Prevalence OR	NA
Bonelli/2003/Italy [23]	18–75	Both (57.4)	21/165	16/165	NA	22/374	18/374	NA	≥3	Patients with non-tumor disease, the same hospital and period	Prevalence OR	Age, gender, smoking, alcohol, center, education and occupation
Lo/2007/Egypt [24]	14–90	Both (57.7)	13/139	13/139	13/139	3/168	3/168	3/168	≥10	Patients with non-tumor disease, the same hospital and period	Prevalence OR	Age, gender, and residence
Anderson/2009/Canada [25]	<80	Both (55.7)	50/371	NA	NA	34/274	NA	NA	>2	Population	Prevalence OR	Age
Kuang/2009/China [26]	61.5	Both (65.7)	43/319	29/319	10/319	29/702	19/702	6/702	≥2	Patients with non-tumor disease, the same hospital and period	Prevalence OR	NA
Maisonneuve/2010/Australia, Canada, the Netherlands and Poland [27]	NA	Both (52.5)	39/679	39/679	39/679	54/1546	54/1546	54/1546	≥10	Population	Prevalence OR	Age, gender, smoking, education, center, and type of interview
Li/2011/USA [28]	NA	Both (57.8)	276/1744	186/1744	108/1744	456/4552	350/4552	229/4552	>2	Population	Prevalence OR	Age, gender, race, education, smoking, alcohol, body mass index (BMI) and study site
Lipworth/2011/Italy [29]	NA	Both (65.9)	76/585	51/585	23/585	108/2077	86/2077	59/2077	≥2	Patients with non-tumor disease, the same hospital and period	Prevalence OR	Age, gender, smoking, BMI, center, year of interview and education
Ben/2011/China [30]	NA	Both (66.9)	96/1055	NA	NA	63/1377	NA	NA	≥2	Patients with non-tumor disease, the same hospital and period	Prevalence OR	Age, smoking, alcohol, family history of pancreatic cancer
Bosetti/2012/Italy and Switzerland [31]	NA	Both (53.4)	57/269	NA	NA	37/615	NA	NA	≥2	Patients with non-tumor disease, the same hospital and period	Prevalence OR	Age, gender, center, year of interview, education, alcohol, smoking and BMI
Henry/2013/USA (1) [32]	≥20	Both (57.6)	28/172	19/172	11/172	42/631	36/631	22/631	≥2	Population	Prevalence OR	Age, gender, race, education, smoking, alcohol and pack-years

a. The cases of the case-control studies are all prevalent cases.

**Table 2:** Characteristics of 6 nested case-control studies.

Author/year/region	Age	Gender (% male)	Case (DM with elected duration/ Non-DM), N			Control (DM with elected duration/ Non-DM), N			Elected DM duration, years	Sampling Scheme [70]					Adjusted factors
			≥2-year	≥5-year	≥10-year	≥2-year	≥5-year	≥10-year		Case selection	Population	Control selection	Assumptions ^a^	Interpretation of reported effect measure	
Hiatt/1988/USA [33]	>25	Both (44.1)	5/44	NA	NA	230/11874	NA	NA	≥5.7	Incidentcases	Closed population	End of follow-up	Rare disease	RR	Age
Attner/2012/Sweden [34]	All ages	Both (53)	51/265	NA	NA	180/2185	NA	NA	≥4	Incidentcases	Dynamic population	Matched on time	NA	RR	Age, sex, domicile
Elena/2013/Finland, USA, Europe and China [35]	37–94	Both (47.8)	141/1339	NA	NA	112/1495	NA	NA	≥2	Incidentcases	Dynamic population	Matched on time	NA	RR	Age, sex, race, education, smoking, alcohol, BMI, PC family history
Henry/2013/USA [32]	≥30	Female	53/237	45/237	29/237	268/2629	218/2629	153/2629	≥2	Incident cases	Closed population	Concurrently	Analysis matched on time	RR	Age, education, smoking, pack-years, BMI
Eijgenraam/2013/Netherlands [36]	55–69	Both (48.2)	23/421	23/421	10/421	127/3835	NA	NA	≥5	Incident cases	Closed cohort	Beginning of follow-up	Censoring unrelated to exposure	RR	Age, gender, smoking, BMI, education, alcohol, PC family history

a. Assumptions were the characteristics of each study, used to evaluate the interpretation of reported effect measure. The evaluations were based on the case selection, population and control selection.

**Table 3:** Characteristics of 20 cohort studies.

Author/year/region	Age	Source and no. of subjects	Gender (% male)	PC cases (DM/ non-DM), N	Person-years (Y)/Numbers (N) of DM/ non-DM	Follow-up (mean years)	Elected diabetes mellitus duration, years	Sampling scheme [70]					Adjusted factors
								Case selection	Population	Non-exposure selection	Assumptions ^a^	Interpretation of reported effect measure	
Adami/1991/Sweden [37]	All ages	Population of Uppsala, 51,008	Both (45.4)	56/-	-	NA	≥5	Incident cases	Closed population	NA	NA	NA	Age, gender
Shibata/1994/USA [38]	All ages	Retired residents, 13,976	Both (NA)	4/38	2410/44787(Y)	NA	>4	Incident cases	Closed population	Beginning of follow-up	Censoring unrelated to exposure	RR	Age, gender, smoking
Chow/1995/Sweden [39]	All ages	Population hospitalized for diabetes, 134,096	Both (47.7)	650/-	901137/-(Y)	6.8 (male)6.7 (female)	≥2	Incident cases	Closed population	Beginning of follow-up	Censoring unrelated to exposure	RR	Age, gender
Jee/2005/Korea (M) [40]	30–95	The Korean population, 829,770	Male	212/529	-	10	≥5	Incident cases	Dynamic population	Matched on time	NA	RR	Age, age squared, smoking, alcohol
Jee/2005/Korea (F) [40]	30–95	Korean population,468,615	Female	60/324	-	10	≥5	Incident cases	Dynamic population	Matched on time	NA	RR	Age, age squared, smoking, alcohol
Stolzenberg-Solomon/2002/Finland [41]	50–69	Male Finnish smokers, 29,048	Male	14/158172	10669/266896(Y)	10.2	≥5	Incident cases	Closed population	Beginning of follow-up	Censoring unrelated to exposure	RR	Age, smoking, physical activity, blood pressure, bronchial asthma history
Khan/2006/Japan (M) [42]	40–79	Japanese inhabitants, 23,378	Male	54/1753	12812.8/176754.5(Y)	NA	≥2	Incident cases	Dynamic population	Matched on time	NA	RR	Age, BMI, smoking, alcohol
Khan/2006/Japan (F) [42]	40–79	Japanese inhabitants, 33,503	Female	69/1554	11176.3/256864.9(Y)	NA	≥2	Incident cases	Dynamic population	Matched on time	NA	RR	Age, BMI, smoking, alcohol
Luo/2007/Japan (M) [43]	40–69	Japanese inhabitants, 47,499	Male	18/110	30273/ 461608(Y)	11	>4	Incident cases	Dynamic population	Matched on time	NA	RR	Age, BMI, smoking, alcohol, physical activity, study area, history of cholelithiasis
Luo/2007/Japan (F) [43]	40–69	Japanese inhabitants, 52,171	Female	6/90	16254/ 555100(Y)	11	>4	Incident cases	Dynamic population	Matched on time	NA	RR	Age, BMI, smoking, alcohol, physical activity, study area, history of cholelithiasis
Stevens/2009/England and Scotland [44]	55.9	Middle-aged women, 129,000	Female	49/1021	-	7.2	>2	Incident cases	Closed population	Beginning of follow-up	Censoring unrelated to exposure	RR	Age, region, socioeconomic status, smoking, BMI, height
Arnold/2009/USA (B) [45]	>30	Population, 48,525	Both (36.3)	35/325	-	20	>2	Incident cases	Dynamic population	Matched on time	NA	RR	Age, sex, smoking, BMI, PC family history, cholecystectomy
Arnold/2009/USA (W) [45]	>30	Population, 1,011,864	Both (43.9)	344/5539	-	20	>2	Incident cases	Dynamic population	Matched on time	NA	RR	Age, sex, smoking, BMI, PC family history, cholecystectomy
Chodick/2010/Israel (M) [46]	≥21	Population, 52,913	Male	25/78	8795/44118(N)	8	≥3	Prevalent cases	NA	NA	NA	Prevalence OR	Age, region, BMI, SES level, use of healthcare services a year prior to index date, history of cardiovascular diseases
Chodick/2010/Israel (F) [46]	≥21	Population, 47,682	Female	23/91	7926/39756(N)	8	≥3	Prevalent cases	NA	NA	NA	RR	Age, region, BMI, SES level, use of healthcare services a year prior to index date, history of cardiovascular diseases
Hemmminki/2010/Sweden [47]	>39	Hospitalized type 2 diabetes patients, 125,126	Both (NA)	133/24324	125126/922796(N)	15	8/9	Incident cases	Dynamic population	Matched on time	NA	RR	Obesity
Wotton/2011/England (1) [48]	≥30	Hospitalized population in National Health Service (NHS) hospitals, 291462	Both (54.6)	24/23779	15898/275564(N)	NA	≥5	Incident cases	Closed population	Beginning of follow-up	Censoring unrelated to exposure	RR	Age in 5-year bands, gender, time period in single calendar years, district of residence
Wotton/2011/England (2) [48]	≥30	Hospitalized population in National Health Service (NHS) hospitals, 192,894	Both (53.1)	5/9770	7771/185123(N)	NA	≥5	Incident cases	Closed population	Beginning of follow-up	Censoring unrelated to exposure	RR	Age in 5-year bands, gender, time period in single calendar years, district of residence
Atchison/2011/USA [49]	18–100	Male veterans from 142 nationwide Veterans Affairs (VA) hospitals, 4,501,578	Male	1656/5983	594815/3906763(N)	10.5	≥2	Incident cases	Closed population	Beginning of follow-up	Censoring unrelated to exposure	RR	Age, time, latency, race, number of visits, diagnoses of alcohol-related conditions, obesity and chronic obstructive pulmonary disease
Yeh/2012/USA [50]	≥30	Adult population of Maryland, 18,280	Both (42.7)	6/63	599/17681(N)	NA	≥2	Incident cases	Closed population	Beginning of follow-up	Censoring unrelated to exposure	RR	Age, the square of age, gender, BMI, smoking, education, hypertension treatment, and high cholesterol treatment
Luo/2013/USA [51]	50–79	Postmenopausal women, 161,808	Female	19/378	27670/1497335(Y)	10.3	≥10	Incident cases	Closed population	Beginning of follow-up	Censoring unrelated to exposure	RR	Age, ethnicity, education, smoking, BMI physical activity, alcohol, energy intake, percent of daily dietary calories from fat, history of hormone therapy

a. Assumptions were the characteristics of each study, used to evaluate the interpretation of reported effect measure. The evaluations were based on the case selection, population and control selection.

The details of quality evaluation for every study are shown in Tables 4–6. The included studies had NOS scores of 4–8.

**Table 4:** Quality of included case-control studies (Newcastle-Ottawa Scale, NOS).

Study	Selection				Comparability		Exposure			Final score
	Case definition adequate	Representativeness of the cases	Selection of controls	Definition of controls	Main factor	Additional factor	Ascertainment of exposure	Same method of ascertainment for cases and controls	Non-response rate	
Cuzick[16]	*	*	0	*	*	*	0	*	0	6/9
Farrow[17]	*	*	*	0	*	*	0	*	*	7/9
Jain[18]	*	*	*	0	*	*	0	*	0	6/9
Bueno de Mesquita[19]	*	*	*	0	*	*	0	*	0	6/9
Gullo[20]	*	*	0	*	*	*	*	*	0	7/9
Lee[21]	*	*	*	*	*	*	0	*	0	7/9
Frye[22]	*	*	0	*	0	0	*	*	0	5/9
Bonelli[23]	*	*	0	*	*	*	*	*	0	7/9
Lo[24]	*	*	0	*	*	*	*	*	0	7/9
Anderson[25]	*	*	*	*	0	0	0	*	0	5/9
Kuang[26]	*	*	0	*	*	*	*	*	0	7/9
Maisonneuve[27]	*	*	*	0	0	0	0	*	0	4/9
Li[28]	*	*	*	*	*	*	0	*	*	8/9
Lipworth[29]	*	*	0	*	0	0	0	*	*	5/9
Ben[30]	*	*	0	*	*	*	*	*	0	7/9
Bosetti[31]	*	*	0	*	*	*	0	*	0	6/9
Henry[32]	*	*	*	0	*	*	0	*	0	6/9

**Table 5:** Quality of included nested case-control studies (Newcastle-Ottawa Scale, NOS).

Study	Selection				Comparability		Exposure			Final score
	Case definition adequate	Representativeness of the cases	Selection of controls	Definition of controls	Main factor	Additional factor	Ascertainment of exposure	Same method of ascertainment for cases and controls	Non-response rate	
Hiatt[33]	*	*	*	*	0	0	0	*	0	5/9
Attner[34]	*	*	*	*	*	*	*	*	0	8/9
Elena[35]	*	*	*	*	*	*	*	*	0	8/9
Henry[32]	*	*	*	*	*	*	0	*	0	7/9
Eijgenraam[36]	*	*	*	0	*	*	*	*	0	7/9

**Table 6:** Quality of included cohort studies (Newcastle-Ottawa Scale, NOS).

Study	Selection				Comparability		Outcome			Final score
	Representativeness of exposure	Selection of the non-exposed	Ascertainment of exposure	Outcome not present at start	Main factor	Additional factor	Assessment ^a^	Follow-up length	Adequacy of follow-up	
Adami[37]	*	*	*	*	*	*	*	*	0	8/9
Shibata[38]	*	*	0	*	*	*	*	*	0	7/9
Chow[39]	*	*	0	*	*	*	*	*	0	7/9
Jee[40]	*	*	*	*	*	*	*	*	0	8/9
Stolzenberg-Solomon[41]	0	*	0	*	*	*	*	*	0	6/9
Khan[42]	*	*	0	*	*	*	*	*	0	8/9
Luo[43]	*	*	0	*	*	*	*	*	*	8/9
Stevens[44]	0	*	0	*	*	*	*	*	0	7/9
Arnold[45]	*	*	0	*	*	*	*	*	*	8/9
Chodick[46]	*	*	*	*	*	*	*	*	0	8/9
Hemmminki[47]	*	*	*	*	*	0	*	*	0	7/9
Wotton[48]	*	*	*	*	*	*	*	*	0	8/9
Atchison[49]	0	*	*	*	*	*	*	*	0	7/9
Yeh[50]	*	*	0	*	*	*	*	*	0	7/9
Luo[51]	0	*	0	*	*	*	*	*	*	7/9

a The outcome assessors were not blinded to the interventions implemented in any of the studies.

### Meta-analysis results

**Fig 2:**
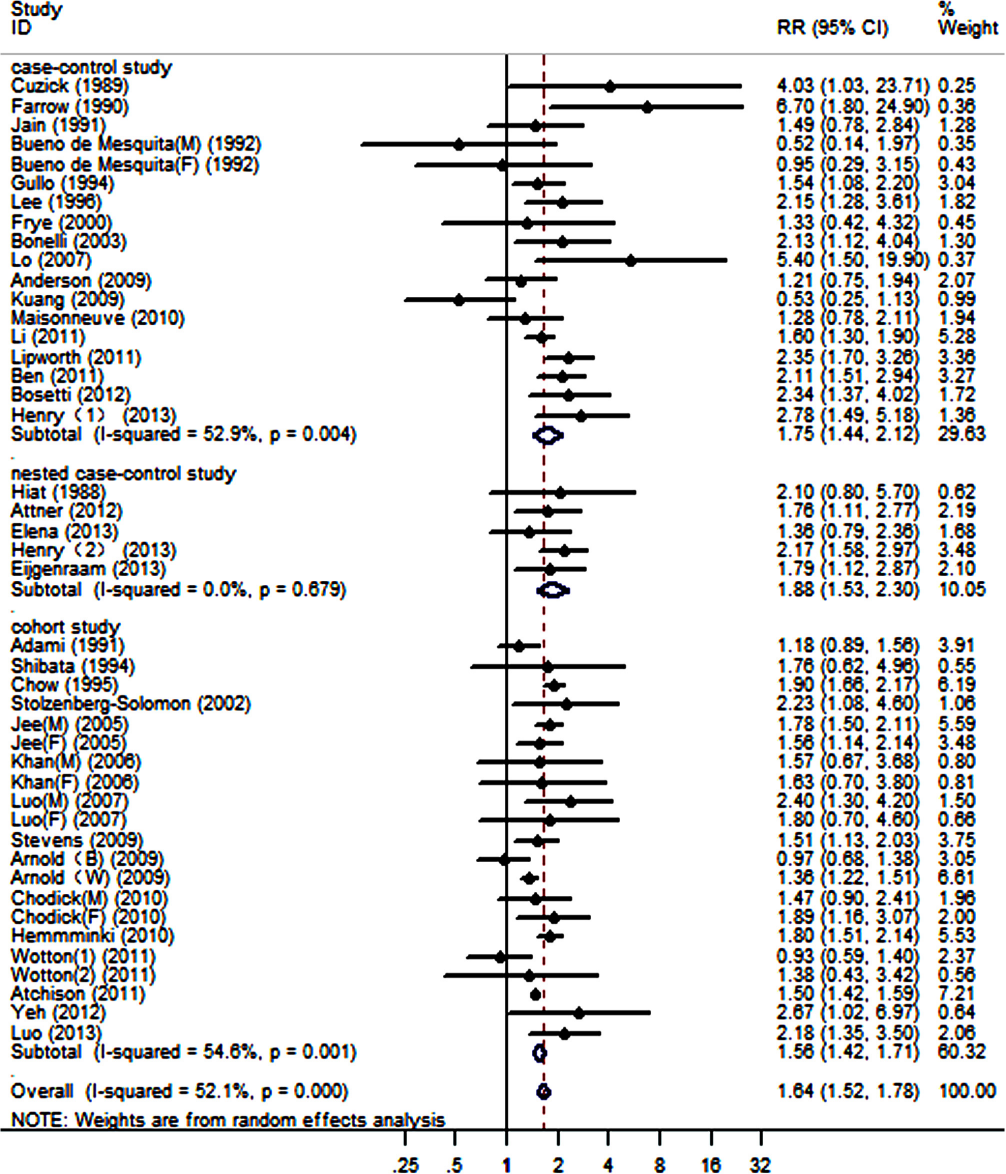
Forest plots of the association between a diabetes duration ≥2 years and the risk of PC.

Pooled RRs for the population with a diabetes duration ≥2 years were calculated using a random-effect model, and the results are shown in Fig 2. For PC patients with long-term diabetes compared with controls without diabetes, we obtained a statistically significant RR of 1.64 (95% CI, 1.52–1.78). We detected heterogeneity among the 44 studies (P<0.001, I^2^ = 52.1%).

**Fig 3:**
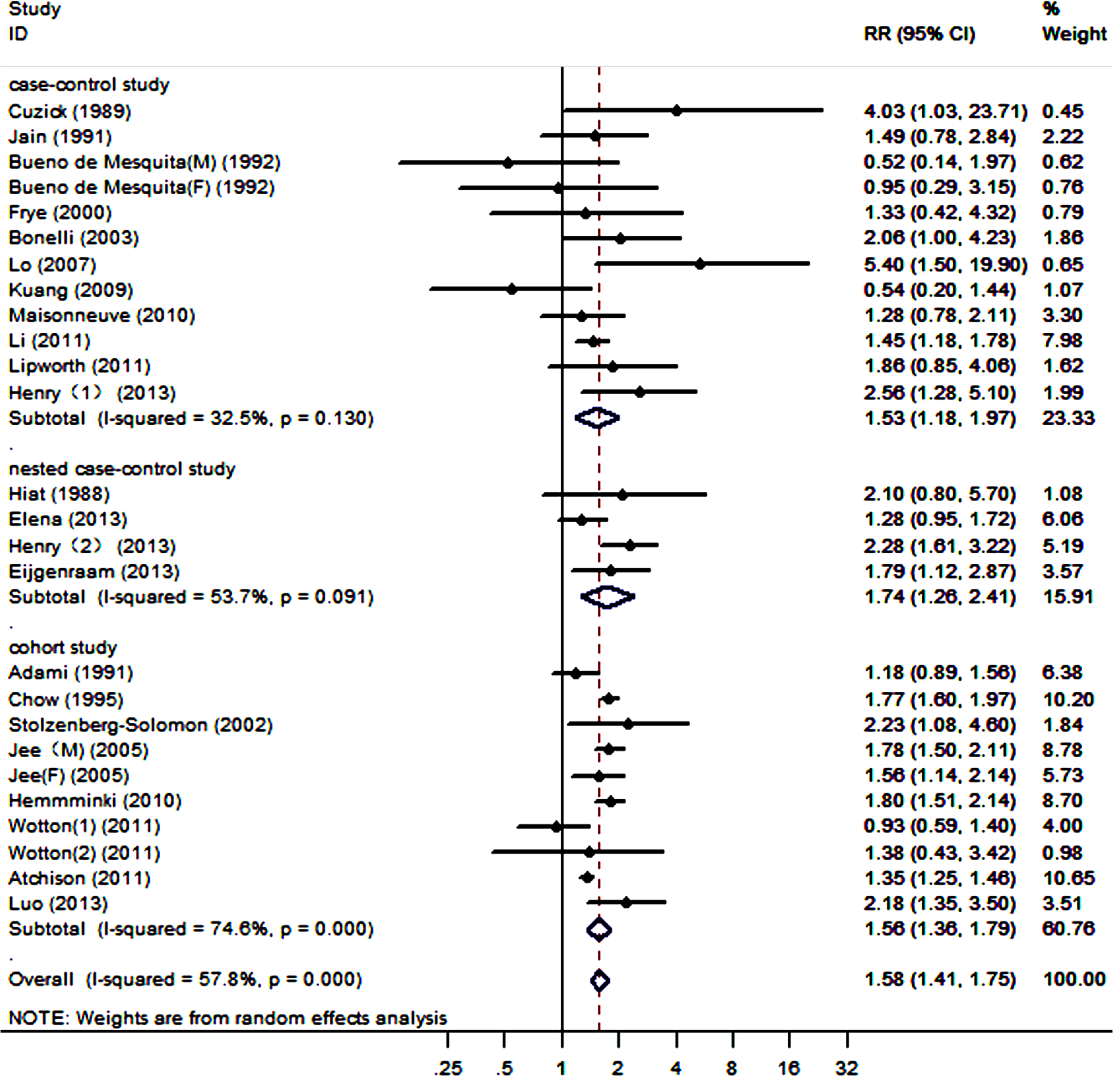
Forest plots of the association between a diabetes duration ≥5 years and the risk of PC.

For the population with a diabetes duration ≥5 years, a significant association among the studies was shown by an average RR of 1.58 (95% CI, 1.42–1.75), which was calculated using a random-effect model (Fig 3). We also detected heterogeneity among the 26 studies (P<0.001, I^2^ = 57.8%).

**Fig 4:**
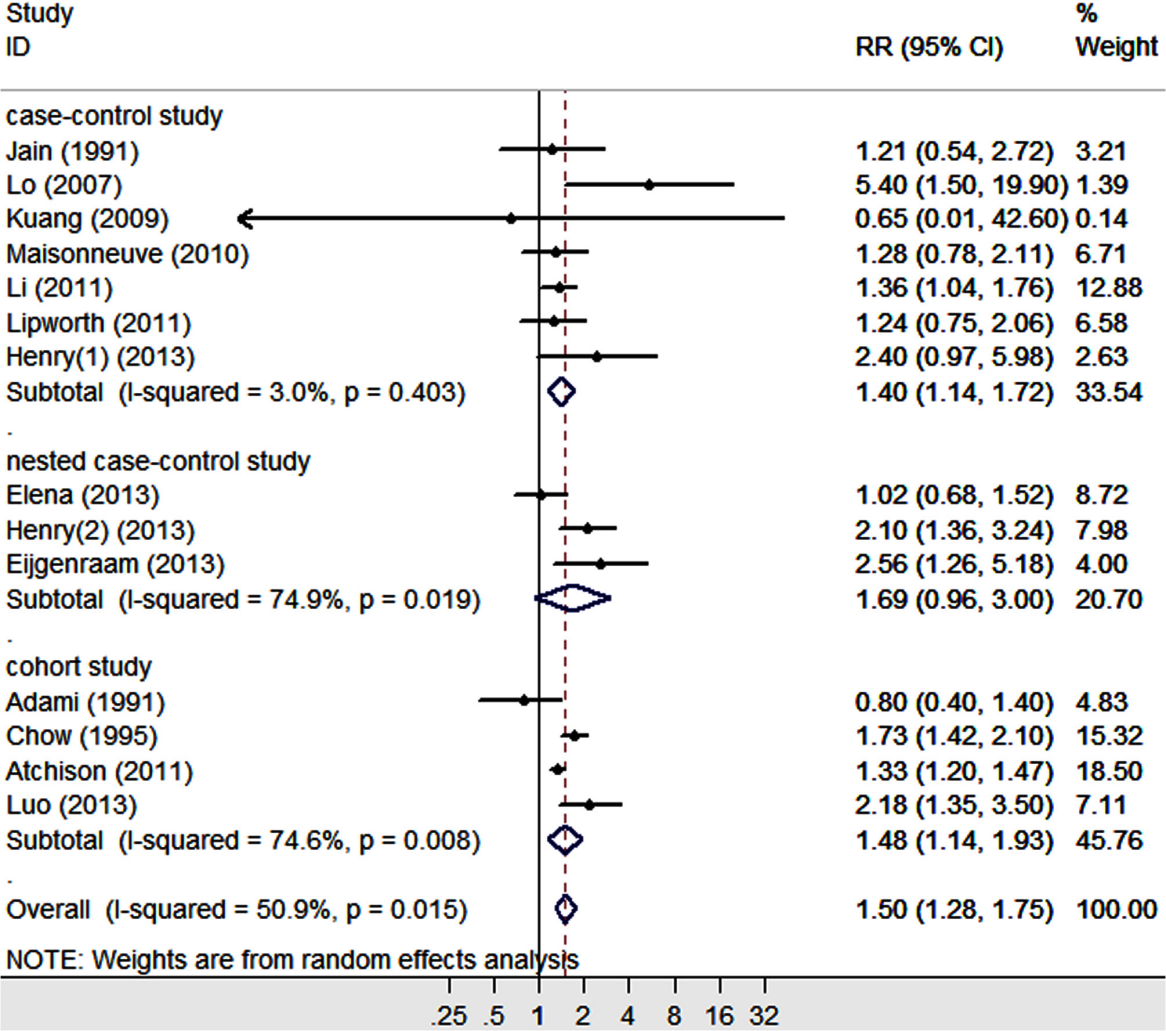
Forest plots of the association between a diabetes duration ≥10 years and the risk of PC.

Additionally, for the population with a diabetes duration ≥10 years, the average RR (95% CI) was 1.50 (1.28–1.75), which was calculated using a random-effect model (Fig 4). Significant heterogeneity was detected among the 14 studies (P = 0.015, I^2^ = 50.9%).

The results from the pooled studies showed that long-term DM was associated with a 1.5- to 1.7-fold increased risk of PC. However, compared with the DM duration category of ≥2 years, the RR of the category of ≥5 years was decreased; with a DM duration of ≥10 years, the RR was further decreased.

### Sensitivity analysis and subgroup

**Fig 5:**
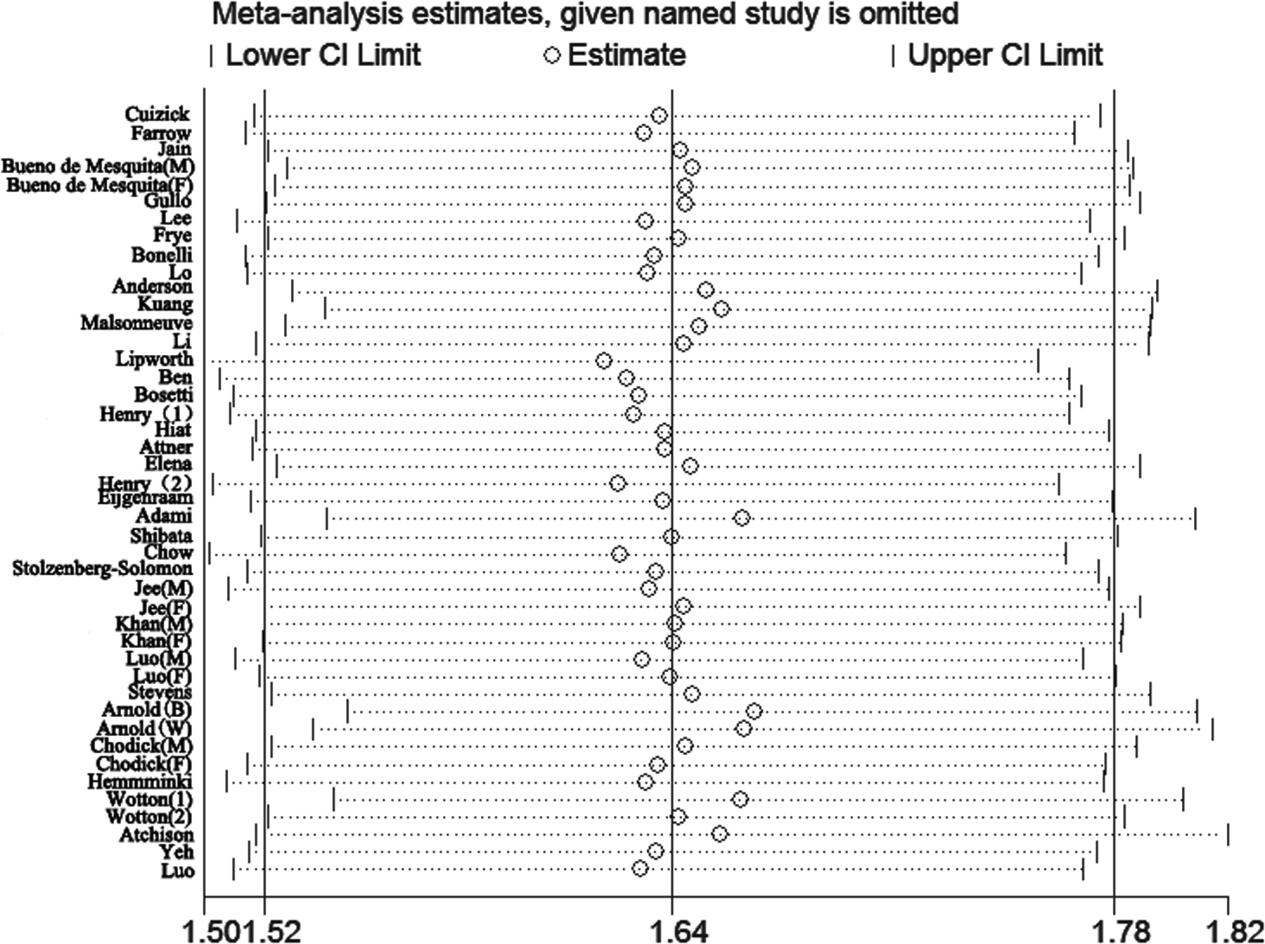
Sensitivity analyses of the studies of patients with diabetes durations ≥2 years.

**Fig 6:**
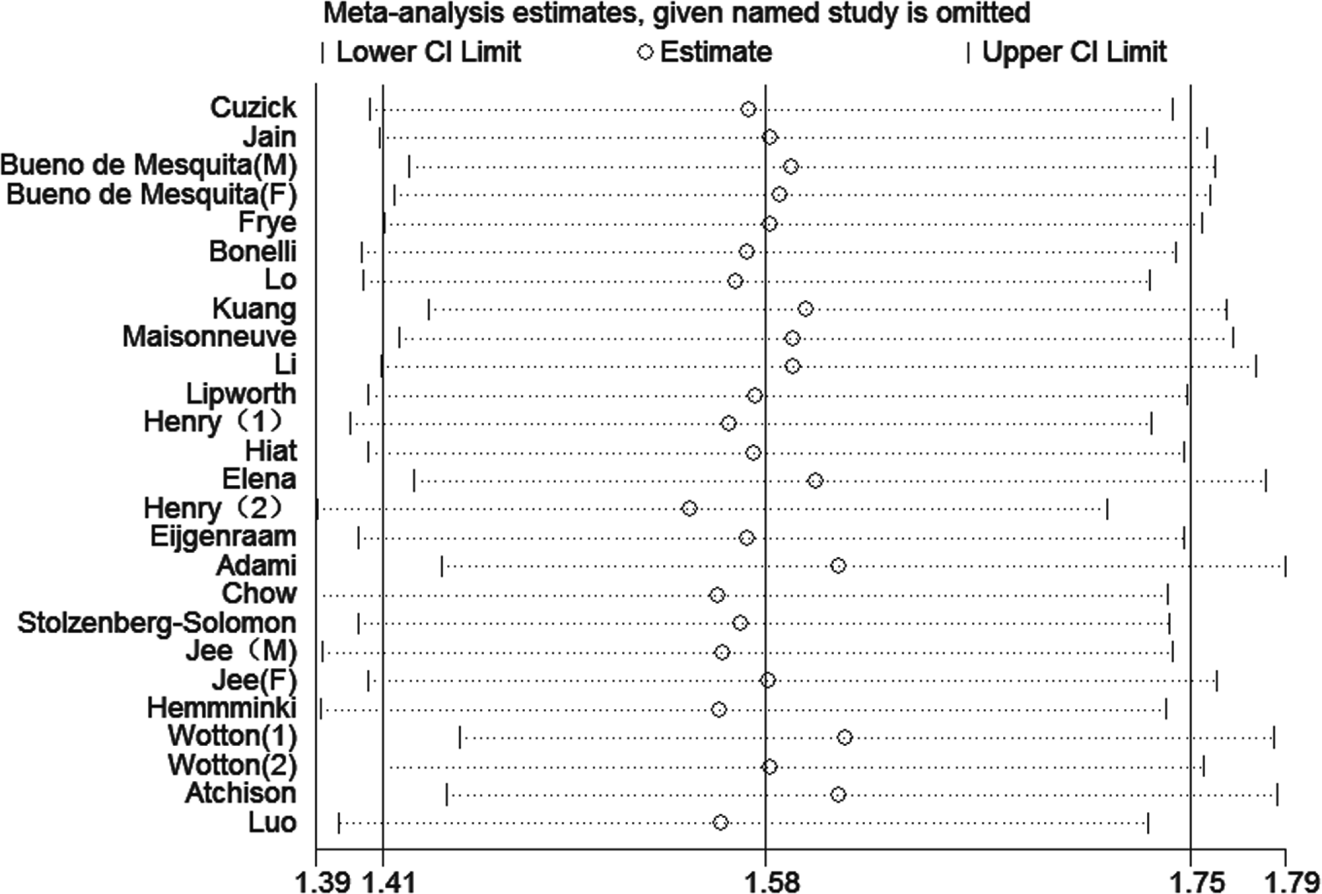
Sensitivity analyses of the studies of patients with diabetes durations ≥5 years.

**Fig 7:**
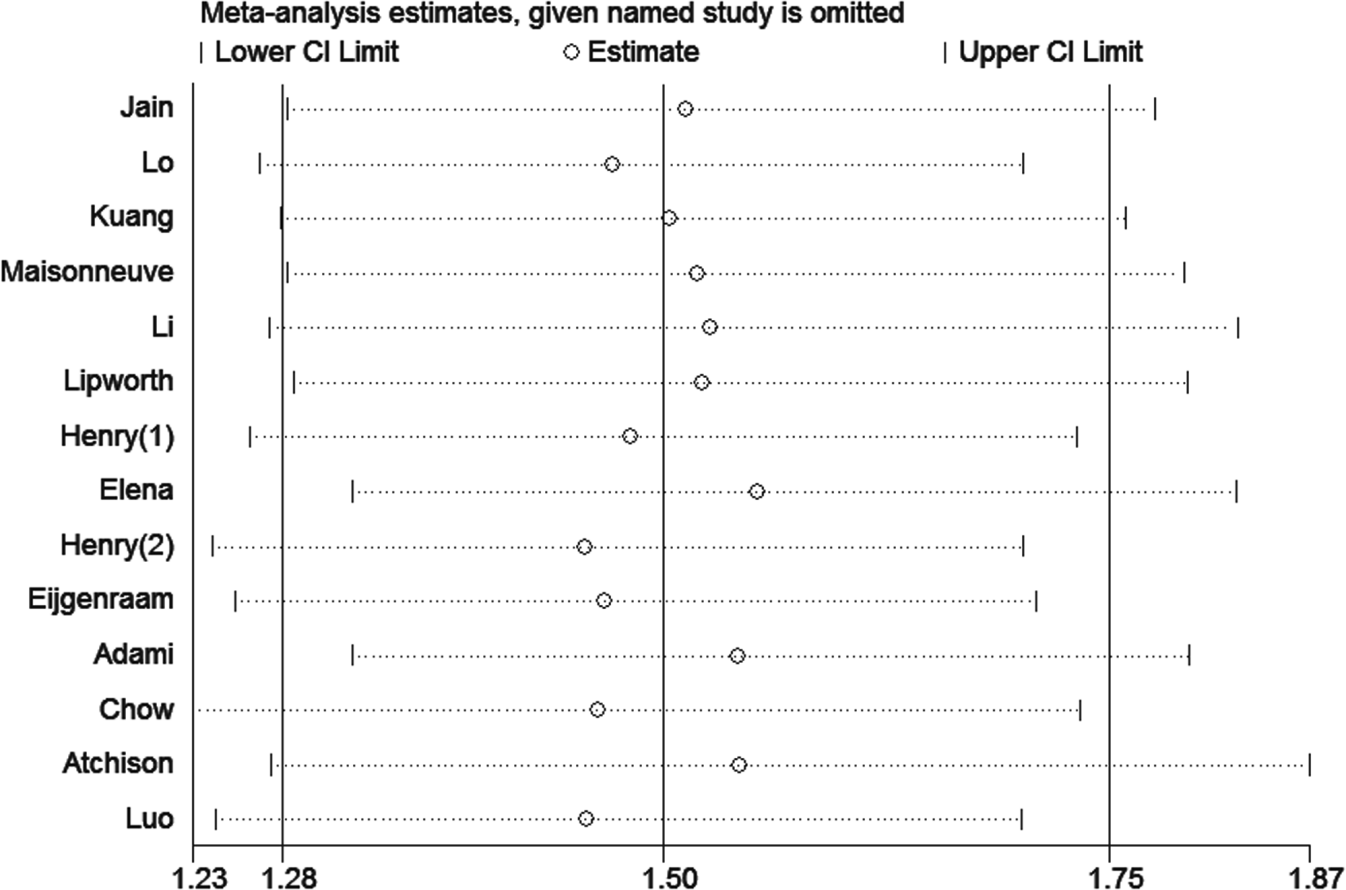
Sensitivity analyses of the studies of patients with diabetes durations ≥10 years.

Due to significant heterogeneity identified among the studies of each DM duration group, sensitivity analysis was performed. The heterogeneity and effect size were calculated by omitting one study in each round. The results suggested that no single study influenced the overall pooled estimates in each group. The results of the sensitivity analysis are presented in Figs 5–7.

Next, we conducted subgroup meta-analyses by gender, study design and region (Table 7). In a total of 7 subgroups among the 3 DM durations, consistent results were found in all subgroups with the pooled effect estimates of the overall analysis; however, inconsistent, nonsignificant outcomes were found when analysis was restricted to 3 nested case-control studies of DM duration ≥10 years. Because I^2^ was not powered to detect heterogeneity with so few included cases, only the P value was used to estimate the heterogeneity in this type of study. According to the I^2^ statistic and P value, significant heterogeneities had a less apparent tendency toward variation.

**Table 7:** Subgroup analysis of included studies.

Criteria		≥2 years					≥5 years					≥10 years			
		No. of studies	Effect model	Heterogeneity (P / I^2^)	RR (95%CI)		No. of studies	Effect model	Heterogeneity (P / I^2^)	RR (95%CI)		No. of studies	Effect model	Heterogeneity (P / I^2^)	RR (95%CI)	
Gender	Male	15	Random	0.003 / 56.9%	1.63 (1.46–1.81)		5	Random	0.017 / 67.0%	1.53 (1.19–1.97)		2	Fixed	0.803 / -	1.33 (1.21–1.47)	
	Female	16	Random	<0.001 / 62.3%	1.72 (1.48–2.01)		5	Fixed	0.239 / 27.3%	1.89 (1.54–2.32)		3	Fixed	0.459 / -	1.96 (1.47–2.63)	
Study design	Case-control study	18	Random	0.004 / 52.9%	1.75 (1.44–2.12)		12	Fixed	0.130 / 32.5%	1.49 (1.27–1.75)		7	Fixed	0.403 / 3.0%	1.40 (1.15–1.70)	
	Nested case-control study	5	Fixed	0.679 / -	1.88 (1.53–2.30)		4	Random	0.091 / 53.7%	1.74 (1.26–2.41)		3	Random	0.019 / 74.9%	1.69 (0.96–3.00)	
	Cohort study	21	Random	0.001 / 54.6%	1.56 (1.43–1.71)		10	Random	<0.001 / 74.6%	1.56 (1.36–1.79)		4	Random	0.008 / 74.6%	1.48 (1.14–1.93)	
Region	Eastern countries	11	Fixed	0.183 / 27.5%	1.75 (1.56–1.97)		1	-	-	-		1	-	-	-	
	Western countries	32	Random	<0.001 / 54.8%	1.61 (1.47–1.76)		22	Random	0.001 / 56.3%	1.56 (1.39–1.75)		12	Random	0.023 / 50.5%	1.47 (1.26–1.71)	

### Publication bias

**Fig 8:**
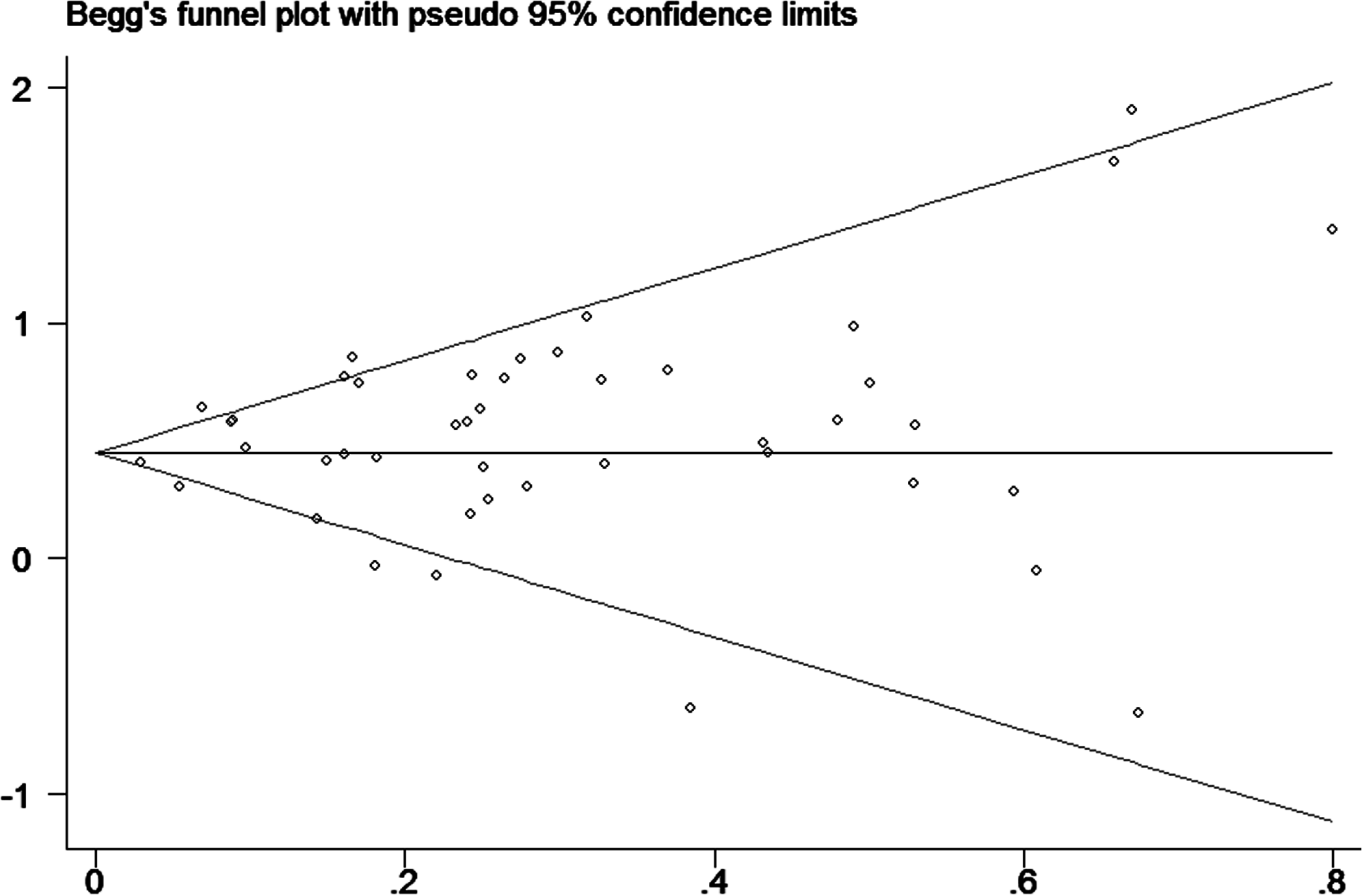
Funnel plot for publication bias of the studies of patients with diabetes durations ≥2 years.

**Fig 9:**
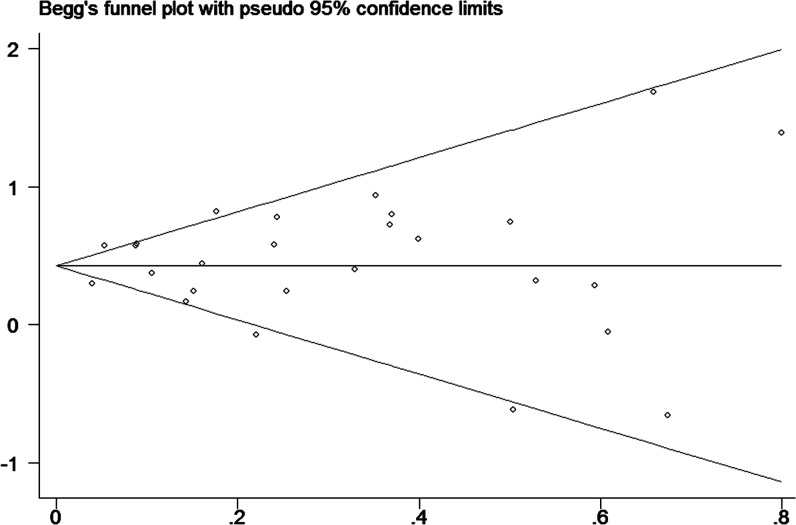
Funnel plot for publication bias of the studies of patients with diabetes durations ≥5 years.

**Fig 10:**
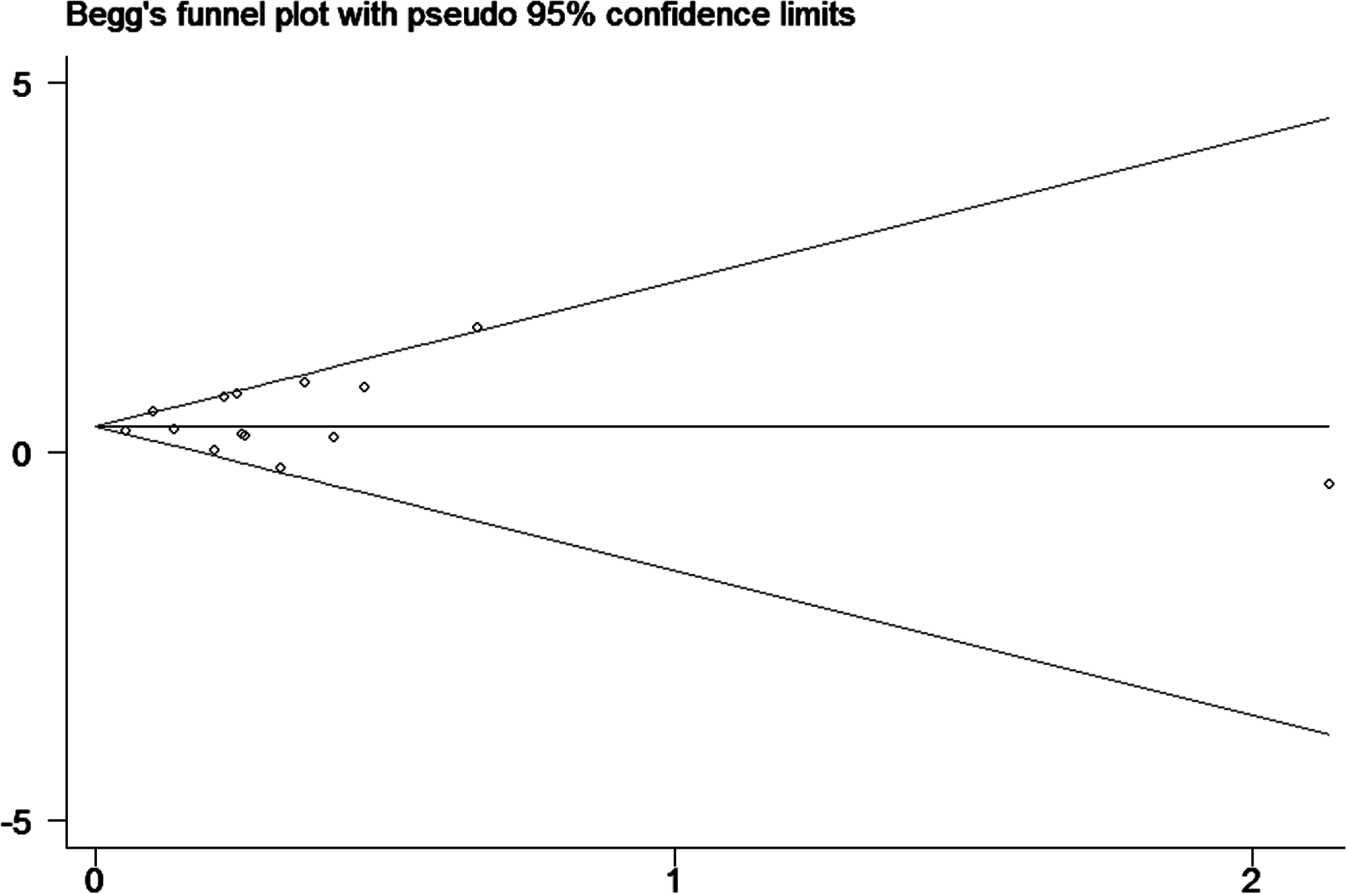
Funnel plot for publication bias of the studies of patients with diabetes durations ≥10 years.

Finally, to assess publication bias of the included studies, we created Begg’s funnel plot. In Begg’s test. Z was 0.13 (P = 0.895) for a DM duration of ≥2 years, 0.40 (P = 0.692) for a DM duration of ≥5 years and 0.33 (P = 0.743) for a DM duration of ≥10 years. We found a low probability of publication bias and small study effects, indicated by P values >0.05 (Figs 8–10).

## Discussion

Our research included 44 studies, which were conducted in different parts of the world and included different populations. This meta-analysis showed a significantly increased risk of PC in diabetes patients who had been diagnosed and followed up for more than 2 years. Using pooled data from the included studies, we found that long-term DM was associated with a 1.5- to 1.7-fold increased risk of PC. The results from the pooled cohort studies and nested case-control studies were consistent with those from the pooled case-control studies. In the subgroup analysis by gender, the risk estimation changed slightly, and gender differences had less influence on the strength of the positive association between diabetes and the risk of PC.

Our findings did not reveal an increased risk of PC with an increasing diabetes duration; in contrast, the RRs were decreased at durations of ≥5 and ≥10 years. The PC risk was negatively correlated with increasing disease duration in the long-term DM category.

Epidemiological studies have reported a bidirectional relationship between diabetes and PC. In previously published systematic reviews [10–12], the studies in which the populations were either diagnosed with new-onset or long-term diabetes were directly pooled. Those results indicated an overall 1.5-fold increased risk of PC among diabetic patients, with a slightly stronger association between diabetes and PC than that observed in our analysis. This finding might suggest that the strength of the association between long-term diabetes and PC development is equal to or slightly weaker than that between new-onset diabetes and PC development.

The mechanism by which diabetes increases the risk of PC development remains controversial. However, this mechanism is likely related to increased insulin-like growth factor 1 (IGF-1) levels, hyperglycemia, insulin resistance and compensatory hyperinsulinemia.

Under physiological conditions, IGF-1 peptides are synthesized mainly by the liver and act as major stimulators of tissue and cellular growth through the induction of proliferation and the inhibition of apoptosis in target tissues [52]. IGF-1 shares 50% amino acid sequence homology with insulin, and as a result, it elicits nearly the same hypoglycemic response [53]. The observed effect of pituitary growth hormone (GH) has been to counteract the action of insulin, leading to insulin resistance in insulin target tissues [54]. The IGF-1-GH axis is believed to play a prominent role in glucose tolerance and type 2 DM.

In particular, studies have shown that IGF receptors are frequently overexpressed in cancer [55], with corresponding changes in the circulating levels of IGF peptides [56–57]. The IGF system and insulin signaling pathway simultaneously play important roles in hyperinsulinemia, insulin resistance and tumor pathogenesis, which are more likely to be the potential mechanisms in a variety of human cancers.

In a Korean study that evaluated a 10-year follow-up period, linear trends in mortality with increasing fasting serum glucose levels were observed for PC. Participants with fasting serum glucose levels of 110 to 125 mg/dL had a significantly elevated risk of PC mortality compared with those with in the reference category (serum glucose levels of 90 mg/dL) [40]. Hyperinsulinemia has been cited as a possible risk factor for PC with the emergence of hyperglycemia. In their research, Li et al. reported that hyperglycemia could lead to oxidative stress, which is caused by an imbalance in reactive oxygen species (ROS) antioxidants. Most patients with PC suffer from diabetes or hyperglycemia, and high glucose can cause ROS production, which can in turn increase the invasiveness of cancer cells [58].

A hyperglycemic state has various indicators; of these indicators, hemoglobinA1c (HbA1c) reflects long-term glycemic control and is a more stable measurement than fasting plasma glucose levels. Cheon et al. undertook research that that a high HbA1c level might be associated with worse survival in patients with advanced PC undergoing antidiabetic treatment [59].

Adiponectin is a hormone that is primarily secreted by adipose tissue; it is inversely correlated with plasma insulin and is reduced in individuals with insulin-resistant conditions such as obesity and type 2 DM [60]. Many case-control and prospective studies have shown that serum adiponectin concentrations are decreased in breast cancer, hepatocellular carcinoma (HCC) and colorectal cancer [61–63]. Bao et al. measured total adiponectin and found that the effects of total plasma adiponectin on PC development might differ from those of high- or low-molecular-weight adiponectin. Interpretation of Bao et al.’s data could warrant the consideration that high/low-molecular-weight adiponectin levels might be more closely associated with the PC risk than total adiponectin [64].

Moreover, large numbers of diabetic patients require life-long drug therapy, which could influence the development of cancer. Insulin, metformin and thiazolidinediones (TZDs) are among the major diabetic therapies shown to improve the control of hyperglycemia through effects on molecular targets such as the insulin receptor and insulin-like growth factor pathways, adenosine monophosphate-activated kinase and peroxisome proliferator-activated receptor γ (PPARγ) [65]. According to recent observational studies, the use of metformin was associated with a reduced risk of PC in patients with diabetes [66]. In addition, TZD demonstrated potent inhibitory effects on the growth of PC cells via the PPARγ-dependent induction of ductal differentiation [67], and the use of insulin glargine was not associated with an increased risk of all cancers or site-specific cancers in Scotland over a 4-year time period [68]. No convincing evidence supports a carcinogenic role of any insulin derivative currently used in therapy. However, the potential contribution of insulin therapy to the hypothetical mitogenic effects of endogenous insulin cannot be fully excluded [69]

Nevertheless, further investigation into the influence of DM drug therapy on cancer development is warranted. Therapeutic strategies should be generally reevaluated, and future research should address these important questions.

This study had several limitations that should be recognized. First, the potential for information and selection biases could not be completely excluded because all of the included studies were observational. Second, some of the included studies did not distinguish between type 1 and type 2 diabetes (although most of the studies included elderly populations and excluded type 1 diabetes); therefore, we could not distinguish the relationships among different types of diabetes, insulin resistance and PC development. Third, the association between long-term DM and the risk of PC is not solely due to DM directly; a theory that DM and PC generation and development share common risk factors has been proposed. More research focusing on these two viewpoints is required. Fourth, none of the included studies considered the role of anti-diabetic drugs in PC development. Finally, in a few of the included studies, the presence of diabetes was self-reported rather than identified from secure records, which could have affected the ascertainment of exposure and distorted the relationships.

In conclusion, our analysis demonstrated that diabetes lasting more than 2 years was associated with an increased risk of PC, after excluding the relationship between PC and new-onset diabetes. Therefore, regular examinations of patients with long-term diabetes could be meaningful for the prediction, early diagnosis and treatment of PC. However, further investigations into the mechanism by which long-term diabetes promotes PC are required.
